# Biomaterials in Menstrual Cup Design: Challenges, Innovations and Future Perspectives

**DOI:** 10.3390/jfb17100527

**Published:** 2026-10-19

**Authors:** Nur Amierah Syafiqah Mohamad, Teishan Puhendhiran, Sakunie Sawai, Foo Kit Cheah, Suhaini Sudi, Helen Lasimbang, Ping-Chin Lee

**Affiliations:** 1 Biotechnology Programme, Faculty of Science and Technology, Universiti Malaysia Sabah, Jalan UMS, Kota Kinabalu 88400, Sabah, Malaysia; nur_amierah_ms24@iluv.ums.edu.my (N.A.S.M.); suhainisudi@ums.edu.my (S.S.); 2 Inno Therapeutics (M) Sdn. Bhd., EmHub Industrial Complex, Selangor Science Park, Kota Damansara, Petaling Jaya 47810, Selangor, Malaysia; research@innotherapeutics.com.my (T.P.); sakuniesawai@innotherapeutics.com.my (S.S.); evancheah@innotherapeutics.com.my (F.K.C.); 3 Department of Obstetrics & Gynaecology, Faculty of Medicine and Health Sciences, Universiti Malaysia Sabah, Jalan UMS, Kota Kinabalu 88400, Sabah, Malaysia; helenlasimbang@ums.edu.my

**Keywords:** biomaterial, menstrual cup, medical-grade silicone, thermoplastic elastomer, rubber

## Abstract

Menstrual cups are an expanding alternative in menstrual hygiene management, positioned at the intersection of biomaterial science, women’s health, and sustainability. Because these intravaginal devices are intended for prolonged, repeated mucosal contact, their safety, functionality, and user acceptance largely depend on material selection and engineering design. This review critically evaluates the biomaterials used in contemporary menstrual cup manufacturing, focusing on medical-grade silicone (MGS), thermoplastic elastomers (TPEs), and natural rubber and compares their physicochemical properties, mechanical behaviour, biocompatibility, sterilisation compatibility, and chemical safety. Beyond material comparison, we draw on clinical, microbiological, and post-market surveillance data to contextualise tissue response, vaginal microbiome stability, and rare but clinically significant adverse events such as toxic shock syndrome. We also examined emerging innovations, including surface and interface engineering, biodegradable elastomers and composite blends, and advances in manufacturing and design. We argue that menstrual cup development is best understood as a multidisciplinary biomaterials challenge integrating polymer chemistry, surface engineering, microbiological safety, regulatory science, and user-centred design. Realising safer, more functional and more sustainable cups will require rigorous biocompatibility testing, long-term studies, and transparent material characterisation.

## 1. Introduction

Biomaterials are substances designed to interact with biological systems and physiological environments to support, improve, or replace biological functions. Biomaterials have been widely used in regenerative medicine, diagnosis and biosensing, cancer therapy, environmental and sustainability innovations, public health and menstrual hygiene products due to their versatile properties. As biomaterials expand across life sciences, their contribution to advancing healthcare and improving global health outcomes has become increasingly evident. Continued development and innovation in biomaterials are thus critical to ensure that these materials remain functional, safe, and biocompatible in contact with human tissue [1,2].

Biomaterials comprise a broad range of material classes, including metallic, ceramic, polymeric and composite systems, each with distinct physicochemical, mechanical and biological properties that determine their suitability for specific biomedical applications [2,3]. Metallic biomaterials such as stainless steel, cobalt–chromium alloys, and titanium alloys are mainly used in load-bearing applications, including orthopaedic and dental implants, owing to their high mechanical strength, ductility, and fatigue resistance [3]. However, their long-term performance in the body is known to be affected by corrosion and by the reactive oxygen species generated on the implant surface, and recent efforts have therefore been directed towards surface engineering approaches, such as antioxidant coatings on titanium-based implants, to improve osseointegration and reduce the risk of implant failure [4]. Ceramic biomaterials include alumina, zirconia and calcium phosphate-based ceramics, which are valued for their bioactivity, wear resistance and chemical stability and are widely investigated for orthopaedic, dental and tissue-engineering applications [3,5]. However, ceramics are brittle in nature, which limits their use in load-bearing sites. Subsequently, composite biomaterials were introduced to overcome the limitations of the individual categories by combining two or more constituents, as demonstrated by biodegradable polymer–ceramic composites that are designed to balance mechanical strength with biodegradability and biological performance [6]. Together, these material classes demonstrate the breadth of biomaterials research and the need to tailor material composition and properties to the requirements of a specific application.

Among the biomaterial classes mentioned, polymeric biomaterials are particularly versatile and widely encountered in daily life, ranging from sutures, catheters and contact lenses to drug delivery systems and personal care products [2]. Unlike metals and ceramics, polymers can be tailored across a wide range of mechanical properties, from rigid to highly elastic. They can be processed into complex geometries at relatively low cost, making them particularly suitable for soft, body-contacting devices. Elastomeric polymers such as silicones and thermoplastic elastomers (TPEs) are of specific interest, as their flexibility, elastic recovery and chemical inertness allow them to conform to soft tissue without causing mechanical irritation, while withstanding repeated cleaning and sterilisation [7]. These are precisely the properties required in menstrual hygiene products, in which the material remains in prolonged and repeated contact with the vaginal mucosa.

Biomaterial-based menstrual hygiene products such as cups and pads are essential to menstrual health. Menstruation, the cyclical shedding of the endometrial lining in women of reproductive age, is a normal physiological process and a marker of reproductive health [8]. Menstrual cups, used to collect menses, are reusable menstrual hygiene products that are small, funnel-shaped medical devices that collect menstrual blood and have been available since the early 1900s. Menstrual cups are used by folding the funnel and inserting the cup into the vagina below the cervix, where the menstrual blood is collected, and the blood flow is not disrupted. They usually hold about 10 to 38 mL of menstrual blood [9]. Menstrual cups are also designed to be used for typically 12 h before being emptied, cleaned and reused. Additionally, as some menstrual cups can last for an extended period, women generally prefer a menstrual cup that is comfortable, economical and environmentally friendly [10]. Ineffective or unsafe products can cause leakage or harm to users, making careful biomaterial selection fundamental to safe hygiene management, shaping reliability, comfort, safety, durability and environmental footprint. With reusable products gaining popularity and menstrual health rising on the global agenda, a systematic understanding of menstrual cup biomaterials is essential to ensure functional, sustainable and equitable menstrual care [11].

This review aims to identify and characterise the most common biomaterials used in current menstrual cup manufacturing, which are medical-grade silicone (MGS), TPE and natural rubber, as well as to trace their evolution since their market introduction. It also evaluates each biomaterial’s biocompatibility, strengths and weaknesses, and identifies current challenges and key research opportunities for improving the safety, functionality, and sustainability of menstrual cups. This narrative review draws on peer-reviewed literature, regulatory documents and patent records identified through PubMed and Google Patents. Searches combined terms such as “menstrual cup,” “biomaterial,” “medical-grade silicone,” “thermoplastic elastomer,” “natural rubber,” “biocompatibility,” and were supplemented by screening the reference list of relevant articles through targeted searches of United States Food and Drug Administration (FDA) 510(k) summaries and the Manufacturer and User Facility Device Experience database (MAUDE) adverse-event database. We prioritised sources published between 2000 and 2026, with earlier foundational and patent literature included where relevant to the historical evolution of menstrual cup materials.

## 2. Fundamentals of Menstrual Cup Biomaterials

The development of menstrual cups spans more than 150 years [12,13], tracing a gradual convergence of material science, biomedical engineering, and women’s health technology. It reflects shifts in material availability, advances in manufacturing and rising user expectations regarding safety, comfort and sustainability.

### 2.1. Early Development of Menstrual Collection Devices

Prior to the advent of modern menstrual products, menstrual management in the early nineteenth century relied almost exclusively on reusable cloth-based absorbents. The conceptual shift toward internal menstrual collection devices emerged in the latter half of the 1800s, marking the earliest menstrual cups as we understand them today. The first recorded invention resembling the modern menstrual cup was developed by S.L. Hockert in the USA in 1867 [12]. The patent, titled Improvement in Menstrual Receiver, described a belt with a string attached to a small rubber pouch. Menstrual fluid was to be collected in the pouch and, like modern menstrual cups, removed periodically to empty it before reinsertion. Further production of the device was halted following patenting, as it was reported that the design lacked practical usability and failed to progress to commercial production. Nonetheless, this design established a foundation in the principle and design of intravaginal menstrual fluid collection devices.

Several further devices were patented over the following seven decades, and together, they show that the intravaginal collection concept was refined repeatedly before a practical product emerged. Mallalieu and Coke’s Catamenial Sack of 1902 retained a rigid corrugated ring at the mouth of the device and suspended a light, flexible, waterproof sack beneath it, together with a buoyant valve-ball that rose with the collected fluid to close the lower opening and limit leakage [14]. Jorgenson’s Catamenial Device of 1913 departed from elastomers altogether, using a cylindrical aluminium body with a hemispherical base that was retained by smooth frictional contact with the vaginal wall; aluminium was selected as light, non-corrosive and resistant to body fluids, but the rigid body could neither be folded for insertion nor conform to the surrounding tissue; thus, this line of development was not pursued further [15]. Across these designs, the collecting element was either externally supported, assembled from several separate parts, or unyielding in contact, and none of them reached sustained manufacture. Section 2.2 examines the transition to a single, self-supporting elastomeric body in the 1930s, which resolved these limitations and produced the form still in use today.

### 2.2. Evolution of Materials in Patented Menstrual Cup Designs

Numerous menstrual cup designs have been patented over the past century, but most introduced incremental refinements, typically aimed at improving insertion, retention, leakage prevention, or comfort rather than fundamental changes in material chemistry. Early designs were built around rubber, the most readily available elastomer of the period, with variations largely confined to its softness, thickness and source. Subsequent patents experimented with rubberised cloth [14], aluminium [15], thermoplastic sheets [16], synthetic resin [17,18], latex and styrenic block copolymer blends [19] and various elastomeric and silicone formulations (Figure 1). To date, subsequent patents have introduced modifications to cup design, functionality and structural features, while the biomaterials used in menstrual cup manufacturing have remained essentially unchanged. Although patent terminology varies, the materials mainly fall within overlapping elastomeric or polymeric families, differing more in processing and formulation than in fundamental chemistry. Several designs nonetheless achieved commercial success and shaped the modern market. Goddard - introduced one of the earliest cups resembling contemporary designs, a one-piece self-supporting form in soft, resilient rubber that required no belts or fasteners in 1932 [13]. However, it was not yet truly reusable The cup, apron, lip and stiffening structures were integrated into a single unit with thickened rims and an embedded coil spring to retain shape and elasticity through repeated folding and insertion over multiple cycles [13]. In contrast, the earlier devices were assemblies: Hockert’s pouch depended on an external belt and string [12], Mallalieu and Coke’s on a supported rubberised cloth sack [14], and Jorgenson’s on a rigid aluminium body [15], each of which introduced either a separate retaining component or an unyielding contact surface. Consolidating the device into one compliant elastomeric body therefore marked the transition from a disposable collection device to a durable, reusable intravaginal product [13].

### 2.3. Biomaterial Selection in Menstrual Cup Manufacturing

In modern manufacturing, biomaterial selection remains one of the most critical determinants of menstrual cup performance and safety [10,25]. As a medical-grade intravaginal device, the cup must meet stringent requirements for biocompatibility, mechanical resilience, chemical stability, and user comfort [26,27]. It must withstand repetitive deformation during insertion, removal, and prolonged wear, while maintaining structural integrity under compressive forces of the vaginal and cervical musculature [10]. Biomaterials intended for such applications must tolerate mechanical stress without rupture or permanent deformation, perform reliably at body temperature (about 37 °C), and remain bioinert to minimise adverse tissue responses [25,27]. Failure to meet these criteria can lead to material degradation, loss of function, or compromised safety [25,27]. These characteristics are important as they directly govern biocompatibility, bioactivity, mechanical behaviour and degradation [28].

Despite extensive historical experimentation with menstrual cup materials, most products on the market today rely on biomaterials introduced decades ago, particularly MGS and TPEs [10,11,26]. This raises a critical question about whether emerging biomaterial and advanced polymer technologies offer meaningful improvements in comfort, biocompatibility, and sustainability, thereby defining the next generation of menstrual hygiene products [29,30].

## 3. Current Biomaterials in Menstrual Cups

Biomaterials are central to both the design and ongoing development of the menstrual cup [10,29]. Advances in material science and bioengineering have progressively improved their comfort, tactile feel and safety [2,7]. Most commercial menstrual cups are manufactured mainly from MGS, TPEs and natural rubber [10,11,26,29].

### 3.1. Medical-Grade Silicone (MGS)

MGS is a polymer built on a siloxane backbone of repeating silicon and oxygen units with methyl-side groups. The physical properties of silicone are determined by the chain length and the degree of cross-linking between chains [31]. It is also the most widely used biomaterial in menstrual cup manufacturing, because its combination of properties is well suited to prolonged intravaginal contact. MGS is inert, non-toxic, and non-irritating and offers a soft, comfortable feel to the user [32]. Its biocompatibility has been confirmed in vitro using MTT cytotoxicity testing under ISO 10993-5, which showed that it is noncytotoxic [33]. The material also tolerates repeated sterilisation by autoclaving, boiling and ultraviolet exposure without loss of integrity and/or function, reflecting its high thermal stability [7] and its resilience and flexible nature, which can contribute to a long shelf-life [34]. Furthermore, MGS is permeable to oxygen, carbon dioxide and moisture, which helps prevent the degradation of the menstrual cups and supports the surrounding tissues, which helps prevent encrustation when the material is exposed to bodily fluids [7]. MGS also has high oxygen permeability, with a permeation coefficient of 900 Barrer [35].

Despite these advantages, MGS has several limitations. Some formulations can release low-molecular-weight siloxanes, which have been associated with endocrine activity [36]. Its high biostability also poses environmental concerns as it is non-biodegradable [37]. There were also isolated cases of contact dermatitis and vaginal irritation with silicone cups, suggesting that prolonged use and individual variation may occasionally cause hypersensitivity [11].

### 3.2. Thermoplastic Elastomers (TPEs)

TPEs are polymers that are made up of an elastomeric domain and a non-elastomeric domain [38]. They combine the natural elasticity of natural rubber with the mechanical strength of thermoplastics. TPEs can be synthesised as copolymers of elastic and non-elastic blocks, as elastomers with dynamic crosslinks or as rubber with thermoplastic blends [39]. This dual character gives TPEs the flexibility and elasticity of elastomers together with the durability and thermostability of thermoplastics, contributing to their wide use as biomaterials. TPE also has wide applications in sectors such as automotive, construction, and electronics [40]. TPEs are typically classified into two main categories, which are thermoplastic olefin (TPO), in which the rubber phase is not crosslinked and thermoplastic vulcanizate (TPV), in which the rubber phase is crosslinked [41]. Other categories of TPEs include thermoplastic natural rubber (TPNR), styrene block copolymer (SBC), polyether block amide (PEBA), and thermoplastic polyurethane (TPU) [37]. These different TPEs offer different advantages due to their tunable mechanical strength, biocompatibility and mechanical resistance. Notably, different TPE subclasses show different profiles, as TPU demonstrates higher mechanical properties and polyamide-based TPEs show enhanced biocompatibility [39].

The use of TPEs in menstrual cups offers several advantages. TPEs exhibit high chemical stability in any biological environment and are mainly resistant to enzymatic degradation, supporting durability in use [34]. TPE also has high mechanical stability, with a tensile strength of 67 MPa [42]. The lack of aeration of the TPE cups limits bacterial biofilm formation. There was significantly reduced Staphylococcus aureus biofilm formation and toxic shock syndrome toxin 1 (TSST-1) in the TPE menstrual cup compared to silicone menstrual cups [43]. However, TPE menstrual cups do have limitations. Some TPE formulations are costly to produce and require complex processes such as dynamic vulcanisation and in situ crosslinking to achieve adequate mechanical properties [41]. TPE cups also tend to have a shorter shelf life of 2–5 years than those made from other biomaterials [29,39]. TPE is also non-biodegradable and can remain in the environment for a long time [29].

### 3.3. Natural Rubber

Natural rubber is derived primarily from natural rubber latex (NRL), a biopolymer (cis-1,4-polyisoprene) extracted from the rubber tree, *Hevea brasiliensis*. It consists of rubber particles surrounded by protein, lipids and long-chain fatty acids that provide a negative charge to the medium and ensure colloidal stability [1]. Its elasticity and resilience make it an attractive material for menstrual cup manufacturing, as it can also reach a high tensile strength of 37 MPa [44]. High tear and tensile resistance contribute to the durability and long shelf life of latex cups [29,44]. Natural rubber is also renewable and biodegradable, offering an environmental advantage over synthetic alternatives [29,45]. Furthermore, NRL resists abrasion, impact, and corrosion, and its insulating properties allow it to disperse heat [45]. NRL is also biocompatible and non-cytotoxic, and it is compatible with short-term heat sterilisation, as long-term heat sterilisation may degrade NRL menstrual cups [29]. NRL is also cost-effective because it is biodegradable [41].

Despite these properties, latex cups have declined in market share compared with MGS and TPEs due to the increased risk of allergic reactions in latex-sensitive users [46]. Latex allergy usually manifests as Type I hypersensitivity, presenting as itching, pruritus and contact urticaria on the skin and sneezing, wheezing and rhinitis in the respiratory system. Latex can also provoke Type IV hypersensitivity, which develops 24–48 h after exposure and manifests as contact dermatitis at the localised contact site [47].

Table 1 summarises the key characteristics of the biomaterials used in manufacturing menstrual cups, showing the advantages and limitations of each biomaterial. In terms of the firmness or feel of the biomaterials, silicone is shown to be the softest material, with the lowest tensile strength of 2–10 MPa [7]. TPE has the highest tensile strength of 67 MPa [42], with also high elasticity and flexibility, and NRL has an intermediate tensile strength of 37 MPa [44]. MGS has the highest biocompatibility compared with TPE and NRL under the ISO 10993-5 standard [48]. MGS is also the highest in oxygen permeability, as the permeability coefficient of MGS is 900 Barrer [35] when compared to TPE 2-50 Barrer [49] and NRL (0.657 Barrer) [50]. In terms of durability, MGS has the longest shelf life (5–10 years), followed by NRL (3–5 years) and TPE (2–5 years) [29,39]. However, MGS has a higher risk of biofilm formation than TPE due to its high aeration [43]. NRL poses a well-established risk of hypersensitivity, especially in latex-sensitive users [46,47]. At the same time, TPE has a rare case of reported allergic reactions, and MGS causes contact dermatitis only in silicone-sensitive users [11,37]. Environmentally, NRL stands out as the only biodegradable biomaterial, as both TPE and MGS are not biodegradable [29,37]. Economically, MGS and NRL have lower production costs than TPE because TPE requires complex processing for dynamic vulcanisation and in situ crosslinking [41]. MGS has the highest market prevalence among biomaterials in menstrual cups [10,11]. In contrast, TPE has lower prevalence due to its costly production and short shelf life despite its favourable low-biofilm characteristics, and NRL has very minimal market prevalence, likely due to its high allergenicity.

## 4. Biocompatibility and Safety Assessments

Although significant advances have improved menstrual cup performance, with MGS and TPE being the most widely used materials [10,11], rigorous biocompatibility and safety evaluation remains essential. The key areas include tissue response and cytotoxicity, microbial safety, and user safety, supported by post-market surveillance. Table 2 summarises the clinical, microbiological and post-market safety studies discussed in this section.

### 4.1. Tissue Response and Cytotoxicity

Tissue-response and cytotoxicity testing are gold-standard components of biocompatibility and safety assessment for biomaterials. Any biomaterial in contact with host tissues can activate the immune system, triggering tissue-material interactions that can lead to a foreign body response (FBR) as the host attempts to manage perceived threats [57]. For menstrual cups specifically, the safety and physiological effects of the constituent biomaterials are typically assessed through physical vaginal examinations, vaginal and Pap smears, and post-void residual (PVR) urine measurements [53,55,56].

Pliable rubber was one of the earliest biomaterials used in menstrual cup design. Early rubber cups such as the Tassette, evaluated in clinical studies from the 1950s–1960s, showed no meaningful changes in vaginal pH, vaginal wall appearance, vaginal smears, or Gram-stain findings, supporting its use as a safe option at that time [53,54]. More recently, Tampax Menstrual Cup was manufactured from a high-performance silicone elastomer (QP1-40 LSR) [56]. Vaginal examination of 62 users revealed no erythema, abrasions, lacerations or ulcerations, with vaginal pH stable at 4.76 against a baseline of 4.38. Users also reported only infrequent burning, itching or stinging. Between 87.4 and 98.4% of users indicated no impact on urination or bowel movements, and PVR measurements showed no evidence of impaired bladder emptying and no urinary tract infections (UTIs) were detected. In the EpiVaginal reconstructed human vaginal tissue model, viability fell to 53.5% during the first 16 h post-use before recovering to 77.7% by 24 h [56]. Under ISO 10993-5, relative cell viability below 70% indicates cytotoxicity [48]. The transient dip below this threshold in the first 16 h, followed by recovery above it within 24 h, is therefore consistent with a mild, reversible cytotoxic response rather than sustained cytotoxicity. This short-term reduction nonetheless warrants further evaluation.

Similar findings were reported for the Softcup, an FDA-cleared menstrual cup made from a proprietary blend of biocompatible polymer [55]. In an evaluation of 406 users across four menstrual cycles, more than 97% reported no adverse effects, no alteration or disruption in vaginal or cervical epithelium and an average vaginal pH of 4.58 [55]. Collectively, historical and contemporary evidence demonstrates that biocompatible polymers used in menstrual cups consistently maintain healthy vaginal physiology and exhibit favourable tissue responses with minimal irritation [53,54,55,56]. However, clinical observations alone may not sufficiently capture potential material-related biological hazards such as those arising from chemical constituents or material-derived substances that may not cause immediate or clinically apparent effects. Therefore, complementary laboratory-based biocompatibility assessments are important to provide a more comprehensive safety profile of menstrual cup biomaterials.

Beyond the reported clinical vaginal examination, a comprehensive biocompatibility evaluation of menstrual cup materials should also consider in vitro cytotoxicity, acute and subchronic systemic cytotoxicity, genotoxicity, and skin sensitisation to assess delayed-type hypersensitivity, where indicated by the intended use and risk assessment. However, most of the data are either not publicly accessible due to their proprietary nature, or direct systemic cytotoxicity data in menstrual cup users remain limited. In a safety assessment of the Tampax Menstrual Cup, the QP1-40 LSR underwent several biocompatibility tests, including in vitro cytotoxicity, acute systemic toxicity, intracutaneous reactivity, and effects upon implantation, but the results remained proprietary [56]. In addition, irritation and sensitisation were supported through read-across from related silicone materials rather than direct testing of QP1-40 [56]. Overall, the proprietary nature of material-related biocompatibility data and limited direct, user-dependent evidence hinder detailed safety evaluation of menstrual cup biomaterials. This highlights the need for more transparent and publicly accessible evidence of biomaterials used in menstrual cup production.

### 4.2. Microbial Safety

Microbial safety is another critical pillar of biocompatibility, complementing tissue-level assessments by evaluating the infection and microbial contamination risks. While biocompatibility focuses on material-tissue interactions, microbial safety examines how a material influences the vaginal microbiome and pathogen growth [47]. Common microbial safety assessments include urinalysis, the whiff test of vaginal secretions for amine odour, microscopic examination of wet mounts and surveillance for *Staphylococcus aureus* colonisation and TSST-1 production. In practice, microbial safety assessments are closely integrated with tissue response and cytotoxicity evaluation, particularly for reusable intravaginal devices such as menstrual cups.

North & Oldham [55] conducted a concurrent microbial safety assessment of the Softcup and reported that use over three successive menstrual cycles did not alter vaginal colonisation by *Staphylococcus aureus*, the etiological agent of TSS. Likewise, cup use did not increase colonisation by microorganisms associated with bacterial vaginosis (*Gardnerella vaginalis* and *Bacteroides* spp.), vulvovaginitis (Candida and other yeasts) or UTI (*Escherichia coli*). Importantly, vaginal Lactobacillus, the normal protective flora, was maintained at healthy levels. Comparable findings were reported by Sica et al. [56] for the Tampax Menstrual Cup, with no clinically meaningful changes in the composition or diversity of the vaginal microbiota and no enhancement of *S. aureus* growth or TSST-1 production. Nevertheless, a rare TSS case has been reported for the Mooncup, an FDA-cleared silicone-based menstrual cup. In February 2012, TSS developed two days after first-time use of a new cup, resulting in a nine-day inpatient stay, with a vaginal swab positive for *S. aureus* [11]. Taken together, these findings indicate that reusable menstrual cups are generally microbiologically safe and do not disrupt the vaginal microbiome, but the occurrence of rare adverse events such as TSS underscores the need for rigorous microbial safety evaluation and post-market surveillance for intravaginal devices. Nevertheless, these findings reflect the microbial safety of the finished product. It may not necessarily indicate the intrinsic antimicrobial activity of the biomaterials themselves.

It is worth noting that MGS and TPE are generally selected based on their chemical stability, flexibility and biocompatibility instead of intrinsic antimicrobial activity [56,58]. The surface properties of silicone, including hydrophobicity and wettability, can influence microbial adhesion; however, these properties do not necessarily prevent microbial colonisation, especially after prolonged exposure to proteins, lipids, and other biological components [58,59] from biological fluids, including menstrual blood, on the surface. Thus, a combination of factors, including material properties, product design, and hygiene and cleaning practices, may influence the microbial safety of menstrual cups. Antimicrobial additives and surface modification may add value in future menstrual cup development by reducing microbial adhesion and biofilm formation. However, their effects on vaginal tissue and the normal microbiota should be carefully evaluated to ensure that enhanced antimicrobial activity does not compromise overall biocompatibility [58,60].

### 4.3. User Safety and Post-Market Surveillance

To ensure long-term safety, real-world effectiveness, and regulatory compliance, post-market surveillance is conducted to detect rare, delayed, and unforeseen adverse effects across larger and more diverse populations, typically underrepresented at the pre-market evaluation stage [61]. The Softcup manufacturer, for example, has conducted FDA-compliant post-market surveillance since the product’s initial distribution in 1996, covering over 100 million cups sold. An average of one complaint per 47,000 cups has been reported, mostly about messiness, difficult insertion and removal and leakage [55]. Between 1996 and 2009, seven complaints were classified as serious, including alleged allergic reactions, vaginal infections, concerns related to TSS, and vaginal wall injury requiring surgical repair. All complaints were submitted to the FDA and recorded in the MAUDE database [55].

Although numerous menstrual cup brands are commercially available worldwide and several studies have reported on their biocompatibility and safety, post-market surveillance data remain relatively limited. A systematic review and meta-analysis encompassing 43 studies on 13 brands provided valuable insights into overall safety and user outcomes [11]. However, long-term real-world safety monitoring data are still sparse. Furthermore, the proprietary nature of material formulations often restricts disclosure of the specific biomaterials used, limiting biocompatibility evaluation at the material level, as most available evidence focuses on the finished product rather than the constituent biomaterials themselves.

Overall, current evidence indicates that MGS, TPEs and related elastomeric polymers used in menstrual cups demonstrate favourable tissue compatibility, minimal cytotoxicity, preservation of normal vaginal physiology, and low risk of microbiological disruption, with few reported adverse events [11,55,56]. Nevertheless, rare complications such as TSS and isolated reports of significant injury highlight that safety cannot be inferred solely from pre-market testing. Robust post-market surveillance, long-term monitoring, and greater transparency regarding material composition are essential for comprehensive biomaterial-level risk assessment. Continued integration of standardised biocompatibility testing with real-world safety data will therefore remain critical to sustaining the safety, regulatory compliance and public confidence of menstrual cup technologies.

## 5. Challenges in Design Considerations for Contemporary Menstrual Cup Biomaterials

Menstrual cups are generally regarded as safe and have gained acceptance as sustainable alternatives to disposable products, with systematic reviews reporting high user acceptability and a low rate of serious adverse events when proper hygiene practices are followed [11]. The materials currently in use are largely biocompatible, yet several material limitations persist and warrant closer scrutiny, as they can affect user comfort, function and safety [10,11]. The fundamental design challenge is to create and maintain a seal within a variable anatomical environment (vaginal canal dimensions, cervical height, pelvic floor tone) while remaining easy to insert, comfortable to wear, and reliably removable without excessive suction or discomfort [10,26]. This “seal–comfort–removability” is primarily determined by the choice of elastomer (silicone, TPE, or latex), formulation, and, often more decisively, geometry including rim thickness profile, cup wall thickness, stem shape, and air-hole design [10]. These challenges therefore extend beyond firmness, sizing and fitting. They also include cleaning, material-associated allergic reactions, chemical safety, and biomechanical compatibility. These challenges are not grounds to dismiss menstrual cups but priority areas for improvement in biomaterial selection, testing, and standardisation.

### 5.1. Geometrical Sizing and Anatomical Fit Variability

Leakage is not a singular failure mode; it occurs through at least three mechanisms [10,11]. First, seal failure occurs when the cup remains partially folded or fails to conform at the rim because of effective rim stiffness and frictional interaction with the mucosa. Second, positional mismatch occurs when the cup sits too low or too high relative to the cervix or when its length or stem causes irritation, reflecting a mismatch between device geometry and individual anatomy [26]. Third, capacity exceedance transpires when the cup volume is inadequate for the flow rate or when filling displaces the air voids needed to maintain the seal [10]. Leakage among cup users ranged from 2% to 31% across studies, attributed to factors such as excessive bleeding, atypical uterine anatomy, and the cup reaching capacity [11]. Because appropriate sizing depends on individual anatomy, firmness preference and flow, selection based on parity or age alone is an inadequate indicator of fit [26].

The “fit” challenge is compounded by inconsistent sizing rationale across the market. MeLuna’s TPE cup system (FDA 510(k) K140626), for example, spans capacities of approximately 10–30 mL across short and regular variants and presents two firmness options at Shore ratings of 40 (Classic) and 50 (Sport), demonstrating that even within a single brand, capacity and stiffness vary in ways that influence both leakage and comfort [52]. United Nations Population Fund (UNFPA)’s technical criteria indicate capacities of approximately 15–40 mL across small, medium, and large procurement sizes [26]. Taken together, these sources imply that the commonly cited “~10–38 mL” range is a realistic market level span, but that capacity is not consistently inferable from external dimensions alone [10].

### 5.2. Mechanical Stiffness and Functional Firmness

A significant gap in contemporary design practice is the lack of globally accepted consumer-relevant mechanical standards. Material type does not reliably predict effective firmness. The compressive load at 50% diameter compression varied approximately fourfold (3.39–13.92 N) across mainstream products, prompting the proposal of categorical firmness bands (very soft to very firm) based on objective compression testing [10]. This matters because the opening behaviour directly influences both leakage risk (insufficient opening leading to inadequate sealing) and discomfort (excessive rigidity resulting in pressure or pain during insertion and wear).

Insertion and removal challenges are often undervalued in materials selection, yet they significantly influence safety and user compliance. Pooled estimates indicate an initial insertion discomfort of ~20%, an insertion failure rate of ~3%, and a use-related discontinuation rate of ~11% [11]. Qualitative evidence consistently points to the need for a “familiarisation phase” across multiple cycles, suggesting that design optimisation should specifically focus on first-cycle usability rather than steady-state performance alone [11]. Procurement guidance correspondingly specifies materials with strong elastic rebound for effortless unfolding and removal, alongside minimum design criteria including smooth surfaces, solid stems (for ease of cleaning), and appropriately sized air holes [26].

Mechanical optimisation should also operate within defined safety and regulatory boundaries. As intravaginal medical devices, menstrual cups are typically subject to ISO 10993 biological assessment standards and quality management systems, such as ISO 13485 and ISO 9001 [26]. These frameworks define acceptable thresholds for cytotoxicity, sensitisation, and irritation, limiting the range of material modifications and surface coatings that can be introduced to improve mechanical properties.

Regulatory classification and manufacturing constraints further shape design choices [62,63]. In the United States, menstrual cups are categorised under 21 Code of Federal Regulations (CFR) 884.5400 as Class II (special controls) and are currently 510(k) exempted with specific limitations [62]. The FDA’s 2025 draft guidance on menstrual products underscores that devices within its scope remain subject to general labelling and quality system requirements [63]. It also highlights the alignment of the US quality system regulation with ISO 13485, effective February 2026, which is particularly pertinent to design controls, documentation, and change management for new biomaterials or coatings [63]. From a materials and design perspective, this indicates that claims of “softness” or “anti-microbial” properties must be substantiated by bench testing, biocompatibility data, and, where applicable, microbiological evidence held within a design history file, and not by user feedback alone [63]. A systematic approach is therefore needed to translate user dissatisfaction into objective engineering responses.

Figure 2a represents this coupling as a triad, with seal reliability, comfort and removability at the vertices and the principal design levers positioned along the edges that link them. Increasing rim and wall stiffness, typical of raising the Shore hardness of the silicone or TPE formulation, improves opening behaviour and seal formation against the compressive forces of the vaginal musculature, but raises the elastic modulus that governs insertion discomfort [10,52]. Increasing surface lubricity reduces shear stress on the vaginal mucosa and eases both insertion and removal but lowers the frictional interaction at the rim on which seal retention partly depends [64]. Strengthening suction and rim profile improves seal reliability while increasing the vacuum that must be released on removal, raising the risk of removal difficulty and vacuum-induced tissue trauma [26,55]. No lever acts on a single objective.

Figure 2b extends the same logic to the full objective set, scoring six single-variable design changes against eight objectives spanning seal reliability, insertion and wear comfort, ease of removal, leakage resistance, biofilm resistance, service life and unit cost. No row is uniformly beneficial. Increasing wall thickness improves seal reliability, leakage resistance and service life while degrading comfort, ease of removal and unit cost. Adding grip texture eases removal but introduces surface features that can harbour biofilm [43,65]. Only a smoother moulder finish offers an unambiguous gain, and it improves comfort and biofilm resistance while leaving the mechanical objectives unchanged [65], so it cannot, by itself, resolve a seal or fit failure.

The practical implication is that menstrual cup design cannot be approached as the sequential correction of individual complaints, since improving any one objective in isolation degrades at least one other. It is a multi-objective problem in which the goal is a balanced compromise rather than an optimum in any single property.

### 5.3. Cleaning, Decontamination, Disinfection, and Sterilisation

While mechanical stiffness contributes to functional performance during use, a cup’s long-term safety and reliability depend on the material’s ability to withstand repeated cleaning and microbial-control procedures without structural or surface degradation. As reusable intravaginal devices, menstrual cups are typically cleaned and, depending on user practice, subjected to heat-based or chemical decontamination or disinfection [26,66]. These routine procedures should be distinguished from formal sterilisation, which destroys all viable microorganisms on the surface, including highly resistant microorganisms such as bacterial spores [67]. Accordingly, boiling, washing, disinfection, decontamination, and sterilisation should not be treated as interchangeable processes [67]. The response of the cup to these respective procedures may also vary according to its material, formulation, surface characteristics, and exposure conditions [68,69].

Inadequate cleaning or decontamination can leave microorganisms and residual biological material on the cup surface after use. This residue, along with microbes, can produce unpleasant odours and, in some cases, infection risks [66]. Standard water rinsing is insufficient to eliminate microbial presence [43]. Persistent colonisation may contribute to conditions associated with menstrual toxic shock syndrome (mTSS). Using a modified tampon sac method, one in vitro study observed greater Staphylococcus aureus proliferation and TSST-1 synthesis with cups than with tampons, attributing this mainly to the aeration associated with cups. A substantial amount of S. aureus biofilm persisted after multiple water washes, reinforcing the recommendation for heat-based decontamination over rinsing alone [43].

Experimental studies have demonstrated effective heat-based decontamination protocols [43,66]. In an in vitro evaluation using the MGS Papperlacup, cleaning the cup with tap water and steeping it for five minutes in boiled water was the most effective method for reducing viable S. aureus, outperforming cold water alone or cold water with soap [66]. In the same study, cleaning a silicone cup with soap and water, followed by steeping in boiled water for five minutes, yielded 0 CFU/cup compared to 2.075 × 10^8^ CFU/cup without cleaning [66]. These findings indicate that brief heat-based decontamination may substantially reduce microbial residue under the tested conditions and may reduce the practical challenges of prolonged boiling. However, these results should not be interpreted as evidence of validated sterilisation, as the study evaluated microbial reduction against only a specific organism and under a defined experimental protocol. Additionally, the findings were obtained using an MGS cup and therefore may not apply to all cup materials and formulations.

More importantly, cleaning, decontamination, disinfection, and sterilisation are not just matters of user practice but are closely linked to the material’s properties [68,69]. While heat-based treatment is most recommended, some users may opt for commercial disinfectants instead [66,69]. Without adequate knowledge or appropriate cleaning reagents, the material may degrade, as some cleaning reagents, such as alcohol, bleach and hydrogen peroxide, contain harsh compounds that may damage the cup surface and create micro-cracks [68,69]. These micro-cracks can compromise mechanical resilience and accelerate crack formation in elastomers [70]. They may also potentially serve as a niche for pathogen accumulation over time [65]. Although silicone elastomers exhibit strong thermal resistance and chemical inertness, surface oxidation and other physicochemical changes may still occur under harsh or repeated chemical exposure [68]. Mechanistically, oxidative exposure converts the near-surface polydimethylsiloxane (PDMS) network into a brittle, silica-like layer with a higher modulus than the bulk; the modulus mismatch under subsequent cyclic deformation drives characteristic surface cracking, which increases effective surface area and roughness and thereby facilitates bacterial attachment [65,68]. Consequently, recommendations for chemical cleaning or disinfection should be considered material-specific rather than universally applicable to all menstrual cups.

One key challenge in reusable menstrual cup design is balancing microbial control with material durability. A material should remain sufficiently stable during repeated cleaning and decontamination throughout its intended service life while maintaining its mechanical and surface properties [68]. At the same time, emerging biodegradable materials must eventually degrade after disposal without compromising performance during use. Long-term studies assessing the cumulative effects of repeated cleaning, heat exposure, chemical disinfection, and formal sterilisation cycles remain limited in the literature, as research has focused largely on clinical safety rather than material ageing [11]. Systematic evaluation of changes in surface morphology, mechanical properties, chemical structure, and microbial adhesion across repeated treatment cycles is therefore needed to establish the long-term durability and safety of both conventional and emerging menstrual cup materials.

### 5.4. Material-Associated Allergic and Hypersensitivity Reactions

Another significant concern relates to material-associated allergic and hypersensitivity reactions. Because of the nature of certain materials used in manufacturing menstrual cups, the polymeric composition of some materials may cause allergies and sensitivities [46,47]. As mentioned earlier, such reactions may also result from poor cleaning or allergic reactions to rubber [46,47]. Reactions to these replacement materials are rare, but they remain clinically meaningful. By 2021, at least three such cases had been reported among menstrual cup users [30]. One case, in 2010, involved a Mooncup user with a reported silicone allergy that required vaginal repair surgery and two cases were associated with the Softcup, which is made from a proprietary blend [55,71]. Although such cases are uncommon, they raise questions about rare individual sensitivities and the effects of long-term exposure. Moreover, the exact compositions of these proprietary polymer blends are not always publicly disclosed, which makes detailed comparison more challenging. MGS, by contrast, is better characterised and behaves more predictably because of extensive research and its established status in the biomedical literature [7,36]. Overall, material transparency is crucial for reporting and evaluating material quality.

### 5.5. Chemical Safety and Leachable Assessment

Beyond allergic responses, chemical safety evaluation must also consider extractable and leachable substances. The vaginal epithelium is highly permeable and is used as a route for drug delivery [72]. Intravaginal exposure to any biomaterial, therefore, carries unique toxicological considerations [73]. The prolonged contact involved in reusable intravaginal devices and repeated monthly use over many years raises questions about long-term safety and chronic exposure [73]. Therefore, detailed chemical safety assessments should be implemented. Chemical safety assessments for a device of this kind follow a defined sequence, formalised in ISO 10993-18 and ISO 10993-17 [74,75]. The base polymer and its additive package, comprising catalysts, crosslinkers, fillers, pigments and processing aids, together with residuals left by incomplete curing, define the pool of substances available to migrate [74]. Chemical characterisation then establishes what is present. Extractables testing determines what can be released under exaggerated conditions, and leachable testing determines what is released under clinical use [74]. The resulting quantities are combined with the exposure scenario, which for a menstrual cup means mucosal contact up to twelve hours per use, repeated across cycles over several years, and evaluated against toxicological thresholds of concern in a formal risk assessment [75]. Each step constrains the next, so a gap at any point limits what the final assessment can conclude.

The Tampax cup, which uses QP1-40 LSR, for example, has undergone thorough chemical safety tests, including extractable testing, Exposure-Based Risk Assessment (EBRA), and biocompatibility assays, under conservative and accelerated conditions to model leaching. It showed no unique or significantly elevated chemical entities, and EBRA showed no safety concerns [56]. This kind of thorough, transparently reported assessment should be standard practice before any materials reach the final stages of production, to ensure product quality and increase user confidence.

### 5.6. Biomechanical Compatibility and User Comfort

One concern prospective users often raise is the possibility of leakage. Because vaginal anatomy varies between individuals, no single menstrual cup fits all users. Still, this is a matter of shape rather than material [10,26]. However, this does not undermine the importance of material composition. While poor fit is a major contributor to leakage, a material’s stiffness and surface characteristics are also important. However, elasticity also affects how well the device conforms to individual vaginal and cervical anatomy. However, these properties are expressed through the rim profile, wall thickness, and surface finish of the completed cup rather than through polymer choice alone. Material choice has been associated with user-reported discomfort and, in rare cases, adverse clinical outcomes [11,55]. In Softcup evaluation, for example, 42 users required physician-assisted removal because of difficulty removing the cup, while other complaints included poor fit (n = 102), leakage (n = 168), and messiness (n = 98) [55]. Other cases include silicone menstrual cups being lodged on the cervix, which required an emergency room visit for cup removal [76], and ureterohydronephrosis, in which the cup compressed and obstructed urinary drainage from the ureter to the bladder [77]. The patient’s symptoms and conditions resolved completely within a month of cup removal [77].

In summary, the challenges discussed in this section are not independent. Anatomical fit variability, the absence of a consumer-relevant firmness standard, material-specific responses to repeated cleaning, rare hypersensitivity and incomplete chemical characterisation each constrain the others: a formulation stiff enough to seal reliably is less comfortable to insert, a surface smooth enough to resist biofilm offers less grip on removal, and a proprietary formulation that performs well in use cannot be assessed at the material level. Conventional menstrual cups nonetheless demonstrate strong overall safety profiles and high user acceptability, and the complications discussed remain rare [11]. Their significance lies less in their frequency than in what they reveal about the difficulty of satisfying several competing requirements simultaneously in a reusable intravaginal device.

## 6. Emerging Materials and Innovations

Recent advances in biomedical engineering have begun to expand the potential functionality of menstrual cups beyond passive fluid collection. Conventional cups are currently manufactured using MGS and TPEs, valued for their biocompatibility, chemical stability, and flexibility. Emerging innovations aim to add functionality to existing cups through four main avenues: bio-based and biodegradable elastomers, surface and interface engineering, applicator systems and multi-material device design, and manufacturing and design innovation. These approaches primarily target four significant barriers: biofouling and cleaning, spillage during removal, insertion and initial usability, and the environmental footprint of the device across its life cycle. Amongst these approaches, surface engineering combined with fluid-management strategies appears particularly promising, whereas recent commercial development has focused more on lubricious coatings and applicator-based delivery systems, and alternative base materials remain at an earlier, largely pre-application stage.

### 6.1. Bio-Based and Biodegradable Elastomers

Sustainability in menstrual care is commonly associated with biodegradability; however, these two factors are distinct [78,79]. A material can be derived from renewable feedstock while remaining resistant after disposal, whereas a biodegradable polymer may be produced from either fossil-derived or renewable resources. This, in particular, is important for a reusable intravaginal device, such as a menstrual cup, as it is held at body temperature with a mildly acidic and moist environment for up to twelve hours at a time, is decontaminated by boiling between cycles and is expected to retain its seal, elasticity and surface integrity over several years of use. Several bio-based and biodegradable elastomer families are candidates in this principle [79]. Their applicability in menstrual-cup design depends on balancing renewable sourcing, controlled degradation, mechanical durability, chemical stability, processability, and biological safety. Three families are most frequently proposed for this role, which include polyhydroxyalkanoates (PHA), polycaprolactone blends (PCL), and bio-derived TPEs. However, none of these materials is currently an established commercial replacement for MGS in menstrual cups, and their applicability remains prospective.

PHAs are polyesters synthesised intracellularly by bacteria from renewable carbon and are among the few genuinely bio-based and biodegradable polymer families available at scale [78]. Their environmental degradation behaviour has been extensively investigated, with degradation proceeding by surface erosion mediated by extracellular PHA depolymerases secreted by bacteria and fungi. It occurs in soil, freshwater, seawater, compost and anaerobic digestion, with reported lifetimes ranging from weeks to months depending on the environment, the degree of crystallinity and the available surface area [80]. This end-of-life profile may benefit menstrual care components, although its relevance to a reusable cup body is less clear. The main challenge lies in material selection, processing, and availability. The more commercially established members of the family, poly(3-hydroxybutyrate) (PHB) and its valerate copolymer, exhibit high stiffness and brittleness rather than elastomeric behaviour, and elastomeric behaviour requires copolymers such as poly(3-hydroxybutyrate-co-4-hydroxybutyrate) or medium-chain-length PHAs (mcl-PHAs) that remain costly and less readily available [78]. More fundamentally, the depolymerase-mediated surface erosion that gives PHAs their environmental advantage has not been adequately evaluated under the conditions a reusable cup imposes, namely repeated wet-heat cycling followed by prolonged mucosal contact, and evidence remains limited regarding the retention of surface integrity over the multi-year service life of such a device.

PCL and PCL-containing blends present a more immediate obstacle for reusable menstrual cup applications. PCL is a semicrystalline polyester that degrades slowly by hydrolysis of its ester linkages, accelerated by lipases and cutinases, with reported lifetimes of approximately two to three years, while its mechanical behaviour is ductile rather than elastic, functioning in blends as a tough phase rather than as the elastomeric component [81]. The key limitation is its melting temperature of approximately 59 to 64 °C [81], which is below the temperature of the boiling-water decontamination mentioned in Section 5.3 as the most effective cleaning protocol, and below autoclave conditions. A cup containing PCL as a major phase may be susceptible to thermal deformation or dimensional instability during thermal reprocessing. PCL and its blends may therefore be suitable for disposable, including applicators and absorbent inserts, where single use precedes disposal and thermal reprocessing is not required.

Bio-derived TPEs represent a promising near-term pathway because they can combine renewable feedstock with mechanical properties suitable for reusable applications without necessarily requiring rapid environmental degradation. TPU elastomers synthesised from castor-oil-derived polyols and related green-carbon building blocks achieve high renewable carbon content while retaining the elastic recovery required for repeated folding. Their hydrolytic and thermal stability can also be adjusted through the choice and ratio of hard and soft segments, with polyether and polycarbonate soft segments generally exhibiting higher hydrolytic resistance than polyester-based counterparts [82]. More broadly, bio-based elastomer chemistries have advanced rapidly, with formulations approaching the mechanical and biocompatibility profiles of conventional synthetic elastomers [79]. However, the limitations remain significant. No bio-based elastomer has an established record of intravaginal mucosal contact, extractable and leachable profiles for this route have not been reported, batch-to-batch variability in bio-derived feedstocks complicates reproducibility, and the regulatory evidence supporting their use in reusable menstrual cups remains limited.

### 6.2. Surface and Interface Innovation

Reusable intravaginal devices are repeatedly exposed to menstrual fluid, a heterogeneous mixture of shed endometrial tissue comprising gland fragments containing stem, epithelial, stromal, and immune cells as well as blood cells, clots, cervicovaginal mucus, and secreted factors such as proteins and extracellular matrix debris [83,84,85,86,87]. Surface properties such as chemistry, wettability and topography critically influence blood adhesion, microbial colonisation, biofilm formation and ease of cleaning [65]. These properties depend not only on the choice of base polymer but also on moulding, finishing, and any coatings applied to the finished cup. Recent advances in biomaterial science have therefore shifted towards surface engineering to enhance functionality without compromising biocompatibility. Three notable approaches are emerging: (i) biopolymer-based blood-solidifying formulations incorporated into the cup, (ii) rhamnolipid biosurfactant coatings for anti-biofilm performance, and (iii) slippery liquid-infused porous surfaces (SLIPS) for self-cleaning.

One proposed strategy incorporates a naturally derived alginate-based powder into the menstrual cup to solidify menstrual blood rather than absorb or contain it [88]. The powder increases fluid viscosity through calcium-mediated ionic crosslinking of high-molecular-weight alginate, a polysaccharide derived from brown algae [89]. It undergoes gelation in the presence of divalent cations such as Ca^2+^ through interactions with guluronate residues [89]. The authors used gelatinised defibrinated blood as both a pad filler and a cup additive, forming a viscous mass that reduces spillage and leakage [88]. Conceptually, this marks a shift in device design. Rather than optimising stiffness and seal, the design modifies fluid mechanics to limit uncontrolled fluid flow from minor seal perturbations or removal. Although the available study excerpt does not fully report gelling kinetics, the qualitative results support feasibility as a leakage-mitigation mechanism. Notably, the authors also incorporated an antimicrobial polymer to inhibit *Staphylococcus aureus*, allowing this formulation to address both leakage control and microbial risk.

A second approach uses rhamnolipids, a biosurfactant produced by *Pseudomonas aeruginosa* that has been identified as a promising surface-active agent with antimicrobial and anti-adhesive properties [90]. The rhamnolipid formulation R89BS has been studied as a coating on MGS to reduce bacterial adhesion and biofilm [91]. When applied to menstrual cups, this coating could limit microbial accumulation associated with repeated use. This aligns with the sustainability goals of reusable products, as biosurfactants are biodegradable and derived from renewable resources. The principal challenge is that the coating’s durability under repeated boiling sterilisation is unknown. This opens a path for future research into covalent immobilisation or multiple coating layers to improve coating longevity while preserving safety and function.

One more advanced strategy involves SLIPS inspired by the Nepenthes pitcher plant [92]. SLIPS resist adhesion across a wide range of fluids, withstand high pressure and can self-clean [92]. Applied to a menstrual cup, SLIPS coating could create a low-adhesion surface that prevents blood fouling and microbial attachment. Building on this principle, a recent design combined a biocompatible silicone oil-infused surface with a biodegradable superabsorbent fibrous tablet of cross-linked alginate, optimised for high absorption (8 mL water or 15 mL blood per g fibre within 8 h) [93]. The tablet retained blood up to the cup’s full capacity, eliminating spillage on removal and making emptying easier. This recontextualises the device as a hybrid system: cup mechanics still govern seal and comfort, while surface lubricity and interior absorbent immobilisation manage hygiene and mess. The lubricant-infused surfaces also prolonged clotting time and exhibited self-cleaning properties, with little residual blood detected on the treated cups and a significant reduction in bacterial adhesion and biofilm formation. However, two limitations apply. Conventional SLIPS generally lack intrinsic antibacterial activity and may defend against bacterial contamination once it occurs [94]. To overcome this, recent SLIPS designs have integrated antimicrobial functionalities that provide both super-repellent and contact-killing antimicrobial performances [94]. Lubricant depletion is a known failure mode of SLIPS in service [92]. The infused oil is gradually lost through shear, dissolution and evaporation, so performance is inherently time-limited, and the depletion rate under repeated boiling and mechanical handling determines whether the approach is viable across a multi-year service life. The biodegradable insert also introduces regulatory and logistical considerations. It may raise “combination product” questions depending on claims and composition, and it increases supply and disposal complexities relative to a single reusable cup [63].

### 6.3. Applicator Systems and Multi-Material Device Design

Simultaneously, device-level usability innovations are advancing through regulatory channels. The FDA 510(k) summary for the Sunny Cup and Applicator (K233361) describes a reusable silicone cup with a lubricious coating, paired with a reusable applicator composed of a nylon body and a TPU tip, both lubriciously coated [64]. Designed for insertion like a tampon applicator, the cup accommodates up to 25 mL and is intended for use for up to 12 h. This aims to alleviate the “folding and placement” difficulties that often accompany first insertion. Nevertheless, it also exacerbates the biomaterials challenge. The additional patient-contacting materials (TPU, nylon, coatings) must meet mucosal-contact biocompatibility criteria, and reprocessing validation must encompass both the cup and the applicator [64].

From a safety science perspective, the paradigm is shifting from “material is inert” towards structured, multi-axis safety evaluation. A four-part assessment framework has been advocated, spanning chemical and extractables evaluation, physical mucosal impact, vaginal microbiota effects, and S. aureus growth and TSST-1 production, indicating that acceptable safety for insertable menstrual devices may require considerations beyond ISO 10993 endpoints [56]. This aligns with the broader regulatory trend in the FDA’s recent draft guidance, which emphasises test planning, quality system controls, and defensible characterisation of materials and coatings, particularly when technical attributes diverge from established predicates [63].

### 6.4. Manufacturing and Design Innovation

Manufacturing innovation is expected to expedite customisation and multi-material designs. LSR is an established material for automated liquid injection moulding in medical-grade applications. DuPont’s Liveo C6-8XX series, for instance, is available in a range of Shore A hardnesses (30–70), is compatible with common sterilisation methods (including autoclaving), and has a typical cure temperature of approximately 150 °C, with curing times contingent on part thickness. This underscores the feasibility of high-throughput silicone cup manufacturing, though it requires substantial capital investment and depends heavily on processing conditions [51]. Additive manufacturing is predominantly used to prototype emergent geometries, such as stiffness gradients, textured grips devoid of biofilm-harbouring lettering, or customised rims. An open-access review of elastomer 3D printing indicates that, although silicone and polyurethane elastomers are appealing for patient-specific medical components, their intrinsic properties pose manufacturing challenges that continue to limit the extensive adoption of 3D printing for end-use elastomer devices [95].

The most credible near-term approach combines incremental biomaterial innovation with device-level validation: coatings and surface treatments that diminish biofouling must be assessed against TSST-1/biofilm issues; applicator systems must demonstrate a net improvement in usability without aggravating removal complications; and any biodegradable or hybrid insert must establish mucosal safety, microbiome compatibility, and real-world acceptability under quality system regulations and procurement criteria. Three constraints apply across all the approaches discussed. Practical feasibility is limited by the fact that every surface strategy reviewed here, including biosurfactant coatings, lubricant-infused surfaces and blood-solidifying additives, has been demonstrated only in vitro, on flat substrates or on a small number of devices, and none has been tested through the hundreds of boiling and folding cycles a cup undergoes across its service life. Economically, the incumbent process sets a demanding benchmark, since liquid injection moulding produces a finished silicone cup in a single automated step, so any added coating, insert or applicator introduces a separate process stage, its own validation burden and a recurring unit cost, in a product category that competes on affordability and on displacing disposables. With respect to long-term safety, these additions carry exposure profiles that the base polymers do not, including depletion of an infused lubricant into the vaginal environment, the fate of a biosurfactant coating as it wears, and the microbiome consequences of a deliberately antimicrobial surface; none has extractables and leachable data for intravaginal exposure, and none has been evaluated over the repeated multi-year exposure a reusable device entails. Table 3 summarises these considerations for each approach. On present evidence, innovations that improve the finished cup without adding a consumable, a coating or a second component, principally improved moulding and surface finish, are the most likely to reach users.

## 7. Conclusions and Prospects

Modern menstrual cups rely predominantly on MGS and TPEs, with natural rubber largely displaced by allergy concerns. This review found that the three materials differ less in biocompatibility, which is broadly comparable, than in the trade-offs each imposes. MGS offers the longest service life, the widest sterilisation window, and the most extensive record of mucosal use, but it is a permanently crosslinked thermoset that is neither biodegradable nor recoverable at end of life. TPEs are reprocessable and have been associated with lower Staphylococcus aureus biofilm formation in one device-level comparison [43], but they have a shorter shelf life and greater formulation complexity. Natural rubber remains the only renewable and biodegradable option and retains high tear resistance [29,44], but its allergenicity has removed it from most markets [46,47].

These advantages are nevertheless bounded at the mucosal interface. No material in current use is free of the potential to provoke local inflammation, and transient epithelial cytotoxicity, contact dermatitis and hypersensitivity have all been reported, indicating that mild and usually reversible mucosal responses may be possible [11,56]. The effect on vaginal flora is limited, with Lactobacillus-dominated communities and vaginal pH preserved in the available studies, but that evidence is short-term and evidence-based rather than material-level, and long-term microbiota stability has not been established [55,56]. These limitations do not undermine the overall safety of the current materials, but they show where further evaluation is needed.

Another finding concerns the evidence itself. Most safety, comfort, and microbial data come from finished products rather than characterised materials, and formulations are often proprietary. Reported differences between cups therefore cannot be attributed to the base polymer alone, since surface finish, geometry, additive package and manufacturing conditions contribute alongside it. Material selection is necessary but not sufficient to determine menstrual cup performance.

Accordingly, the design and characterisation of intravaginal biomaterials is best treated as a multiparametric engineering problem rather than a search for a single optimal material. Mechanical stiffness, volumetric capacity, polymer chemistry, gas permeability, surface lubricity and friction, sterilisation tolerance, leachable profile and environmental footprint act as simultaneous constraints and conflicts. The stiffness that secures a seal aggravates insertion discomfort, and the hydrolytic stability that gives long service life precludes biodegradability. Formulation development would therefore benefit from formalising a multi-objective treatment. Multi-criteria decision-making and rank aggregation approaches, which combine weighted criteria or aggregate pairwise comparisons into a consensus ranking [97,98], offer a structured way to identify formulations with balanced overall performance rather than optimising any single property. Realising safer, more functional and more sustainable cups will require this alongside rigorous biocompatibility and durability testing, transparent material characterisation, and user-centric design within a clear regulatory framework.

## Figures and Tables

**Figure 1:**
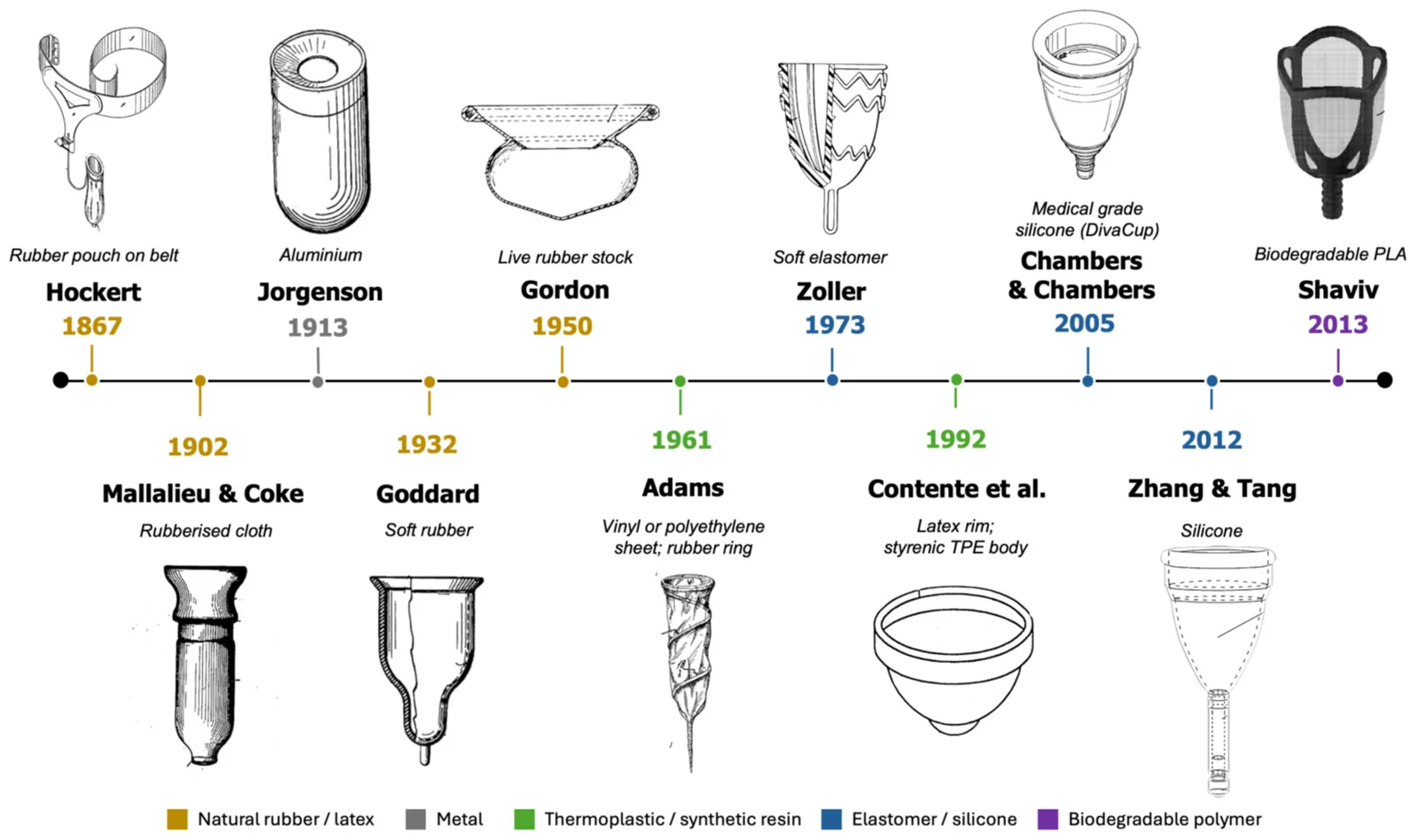
Historical evolution of menstrual cup materials and designs, 1867–2013, across eleven representative patents. Entries are positioned by the year of filing and, in chronological order, are: Hockert, 1867 [12]; Mallalieu and Coke, 1902 [14]; Jorgenson, 1913 [15]; Goddard, 1932 [13]; Gordon, 1950 [20]; Adams, 1961 [16]; Zoller, 1973 [21]; Contente et al., 1992 [19]; Chambers and Chambers, 2005 [22]; Zhang and Tang, 2012 [23]; and Shaviv, 2013 [24]. The device drawings are reproduced by the authors from the cited patent specifications, which are official publications of the United States Patent and Trademark Office and the World Intellectual Property Organisation and are not subject to copyright restrictions. The composite figure, including its layout, material annotation and colour coding, was compiled by the authors.

**Figure 2:**
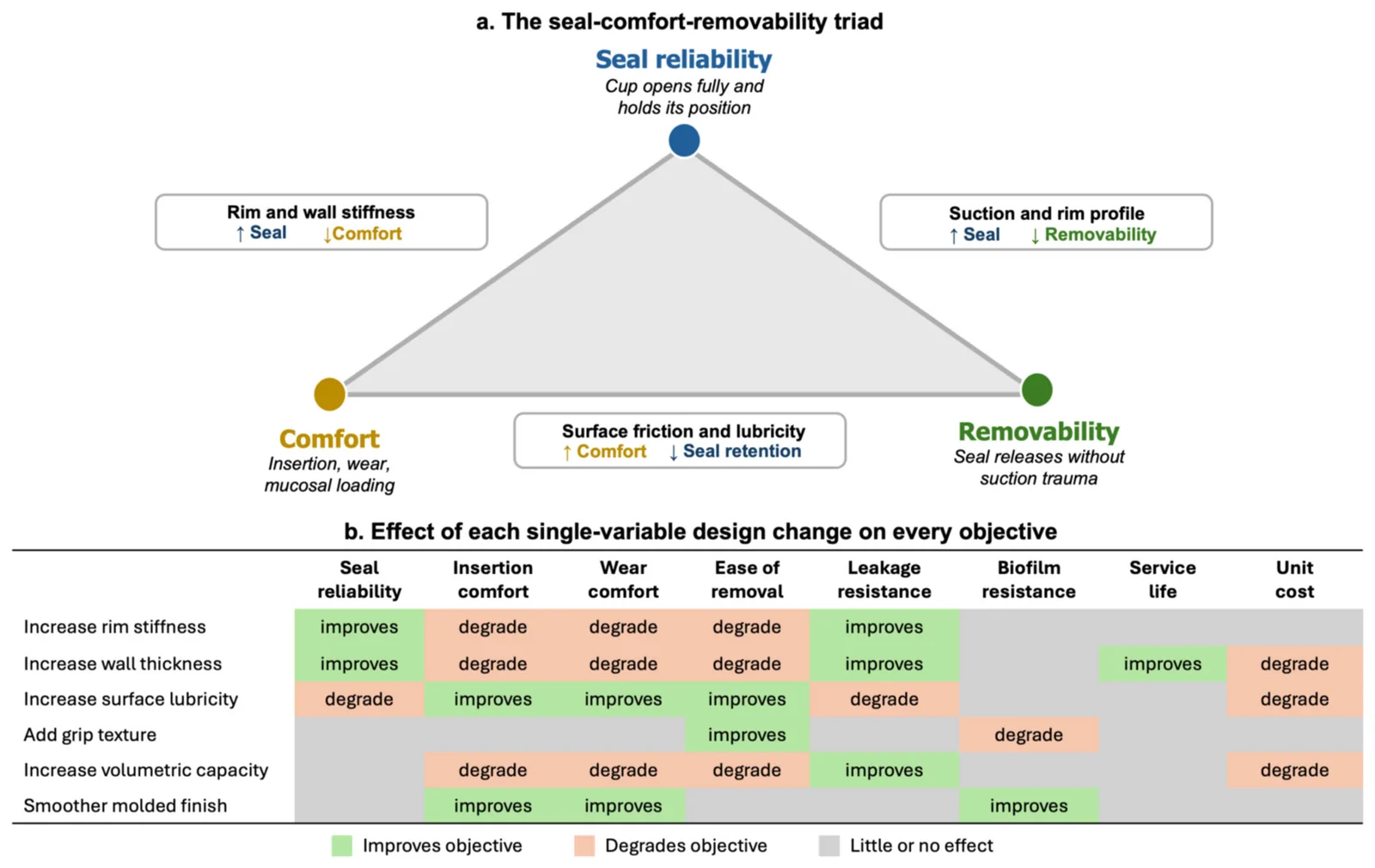
Multi-objective design framework for menstrual cups. (**a**) The seal–comfort–removability triad. Each edge of the triangle carries the design lever that couples the two objectives it joins, and within each lever the objective gained, and the objective spent are coloured to match their vertices, so the cost of every adjustment is legible at a glance. No lever acts on one objective alone. (**b**) Effect of each single-variable design change on the full objective set. No row is uniformly beneficial, which is the defining characteristic of a multi-objective design problem and the reason single-variable optimisation cannot yield a globally improved device. Both panels were constructed by the authors and summarised the directional relationships described in Section 5.1 and Section 5.2. Entries indicate the expected direction of effect rather than measured magnitudes.

**Table 1:** Summary of key characteristics of current biomaterials used in menstrual cups.

Material	Medical-Grade Silicone	Thermoplastic Elastomer	Natural Rubber
Composition	Siloxane backbone of silicon, oxygen and methyl side groups; an array of PDMS monomers of varying lengths, with high crosslinking and strong, polar Si-O bonds [43]	Made of an elastomeric and non-elastomeric domain [39]	NRL consisting mainly of isoprene (cis-1,4-polyisoprene) extracted from *Hevea brasiliensis* [1]
Hardness (Shore A)	30–70 (device-grade LSR grades) [51]	40 (Classic) and 50 (Sport) in commercial cups [52]	Not specifically reported for menstrual cup rubber.
Feel and tensile strength	Soft; 2–10 MPa [7]	Flexible and elastic; 67 MPa [42]	Elastic and resilient; ≈37 MPa [44]
Biocompatibility	Highly biocompatible under ISO 10993-5 standard [33]	Biocompatible under ISO and FDA standards [29]	Biocompatible but less biocompatible than MGS and TPE [29]
Sterilisation compatibility	Compatible with autoclaving and UV sterilisation [7]	Compatible with boiling and autoclaving [29]	Compatible with boiling/heat in the short term but prone to degradation [29]
Oxygen permeability	High; permeability coefficient ≈ 900 Barrer [35]	Low, ≈2–50 Barrer [49]	Lower than both MGS and TPE; ≈ 0.657 Barrer [50]
Durability	High shelf life; 5–10 years [29]	Low shelf life; 2–5 years [29]	High shelf life; 3–5 years [29]
Biofilm	High risk, due to high oxygen permeability [43]	Low risk, due to low oxygen permeability [43]	Possible risk
Allergy	May cause contact dermatitis in silicone-sensitive patients [37]	Rare [29]	Risk in latex- sensitive users [46]
Environmental footprint	Non-biodegradable; high stability [37]	Non-biodegradable; may contribute to microplastics [29]	Biodegradable and renewable [29]
Relative cost	Low relative to TPE [41]	High (dynamic vulcanisation, in situ crosslinking [41]	Lower relative to TPE [41]
Market prevalence	Dominant material in commercial menstrual cups [10,11]	Less widely used than MGS	Minimal, due to allergy concerns

**Table 2:** Overview of menstrual cup biomaterials, safety assessments, and clinical outcomes.

Brand	Biomaterial	Type of Assessment	Outcome	Reference
Tassette (n = 147)	Pliable rubber	Vaginal pH, vaginal wall, vaginal smears, Gram stains	No meaningful changes were noted.	[53,54]
Softcup (n = 406)	Proprietary blend of soft biocompatible polymer compounds	Physical examination, Pap smear, urinalysis, vaginal pH, whiff test of vaginal secretion for amine odour, microscopic examination (wet mounts)	No adverse effects, no alteration or disruption in vaginal or cervical epithelium. 91% of the women harboured lactobacilli, *G. vaginalis*, group B *Streptococcus*, *Enterococcus*, *S. aureus*, *E. coli*, yeast, and *Bacteroides* spp. at frequencies considered normal and consistent. No changes in normal flora were found.	[55]
		Post-market surveillance	One complaint per 47,000 cups sold Minor: Messiness, difficult insertion, difficult removal, leakage Major: Difficult removal, vaginal infection, alleged allergic reaction, vaginal infection, TSS concern, cup worn through the vaginal wall (damaging artery), requiring surgical repair	[55]
Mooncup (n = 550)	Silicone	Toxic Shock Syndrome (TSS)	TSS two days after using the first and new Mooncup, resulting in a 9-day inpatient hospital stay Vaginal swab positive for *S. aureus*	[11]
Tampax Menstrual Cup (n = 62)	QP1-40 LSR	Vaginal examination, pH, PVR, self-reported comfort, microbiota swabs	No clinically relevant pH changes; No meaningful adverse effects	[56]
		In vitro microbial growth, TSST-1 production	No enhancement of *S. aureus* growth or TSST-1 production attributable to cup	[56]

**Table 3:** Emerging materials, surface strategies and manufacturing approaches for menstrual cups, with the barrier each addresses, its principal advantage and limitation, and its current stage of development.

Approach (Example)	Barrier Addressed	Principal Advantage	Principal Limitation	Stage of Development
Bio-based and biodegradable elastomers (Section 6.1)				
PHAs (PHB, PHBV, P(3HB-co-4HB), mcl-PHAs) [76,77]	Environmental footprint	Fully bio-based; degrade by depolymerase-mediated surface erosion in soil, freshwater, seawater, compost and anaerobic digestion, over weeks to months	PHB and PHBV are stiff and brittle rather than elastomeric; elastomeric copolymers remain costly and less available; surface integrity under repeated wet-heat cycling and multi-year mucosal contact not evaluated	Prospective; not applied to menstrual cups
PCL and PCL blends [78]	Environmental footprint	Established biodegradable polyester; hydrolytic and enzymatic degradation over approximately two to three years	Melting temperature of approximately 59–64 °C lies below the boiling-water decontamination identified in Section 5.3; ductile rather than elastic	Unsuitable for the cup body; possible for disposable components
Bio-derived TPEs (castor-oil-derived TPU) [79,80]	Environmental footprint	High renewable carbon content with elastic recovery retained; hydrolytic and thermal stability tunable through hard- and soft-segment chemistry	Renewable origin confers no end-of-life advantage; no record of intravaginal mucosal contact; feedstock batch variability; no regulatory precedent	Most credible near-term bio-based route
Surface and interface engineering (Section 6.2)				
Alginate blood-solidifying powder [86,87]	Spillage on removal; leakage	Raises fluid viscosity by calcium-mediated ionic crosslinking, reducing spillage; antimicrobial polymer co-incorporated to address microbial risk	Gelation kinetics incompletely reported; introduces a consumable into a reusable device	Proof of concept, in vitro
Rhamnolipid biosurfactant coating (R89BS) [88,89]	Biofouling and cleaning	Reduces bacterial adhesion and biofilm formation on MGS; biodegradable and renewably sourced	Coating durability under repeated boiling unknown; antimicrobial claims require substantiation within a design history file	Proof of concept, in vitro
SLIPS [90,92]	Biofouling and cleaning; spillage	Low blood fouling and self-cleaning; prolonged clotting time and reduced bacterial adhesion and biofilm formation	No intrinsic antibacterial activity unless functionalised; lubricant depletion through shear, dissolution and evaporation limits service life	Proof of concept, in vitro
Hybrid cup with superabsorbent alginate insert [91]	Spillage on removal	Retains blood to the full capacity of the cup, eliminating spillage on removal and easing emptying	Introduces a disposable component; may raise combination-product questions; increases supply and disposal complexity	Proof of concept
Device design and manufacturing (Section 6.3 and Section 6.4)				
Lubriciously coated cup with reusable applicator [96]	Insertion and initial usability	Addresses the folding and placement difficulty that accompanies first insertion	Additional patient-contacting materials (TPU, nylon, coatings) must each satisfy mucosal biocompatibility; reprocessing validation must cover both components	Commercial; FDA 510(k) cleared
LSR injection moulding [93]	Manufacturing consistency	High throughput; Shore A 30–70 range; autoclave compatible; tight dimensional and surface control	Substantial capital investment; strong dependence on cure conditions; unsuited to small-batch or customised production	Established industrial standard
Additive manufacturing of elastomers [94]	Anatomical fit variability	Enables stiffness gradients, textured grips without biofilm-harbouring lettering, and customised rims	Intrinsic properties of silicone and polyurethane elastomers continue to limit adoption for end-use devices; layer roughness is a biofilm liability in this application	Prototyping; limited end-use

## Data Availability

No new data were created or analyzed in this study. Data sharing is not applicable to this article.

## Abbreviation

The following abbreviations are used in this manuscript:

CFR	Code of Federal Regulations
CFU	Colony-forming unit
EBRA	Exposure-based risk assessment
FBR	Foreign body response
FDA	United States Food and Drug Administration
ISO	International Organisation for Standardisation
LSR	Liquid silicone rubber
MAUDE	Manufacturer and User Facility Device Experience database
mcl-PHA	Medium-chain-length polyhydroxyalkanoate
MGS	Medical-grade silicone
mTSS	Menstrual toxic shock syndrome
MTT	3-(4,5-Dimethylthiazol-2-yl)-2,5-diphenyltetrazolium bromide
NRL	Natural rubber latex
P(3HB-co-4HB)	Poly(3-hydroxybutyrate-co-4-hydroxybutyrate)
PCL	Polycaprolactone
PDMS	Polydimethylsiloxane
PEBA	Polyether block amide
PHA	Polyhydroxyalkanoate
PHB	Poly(3-hydroxybutyrate)
PHBV	Poly(3-hydroxybutyrate-co-3-hydroxyvalerate
PVR	Post-void residual (urine volume)
SBC	Styrene block copolymer
SLIPS	Slippery liquid-infused porous surface
TPE	Thermoplastic elastomer
TPNR	Thermoplastic natural rubber
TPO	Thermoplastic olefin
TPU	Thermoplastic polyurethane
TPV	Thermoplastic vulcanizate
TSS	Toxic shock syndrome
TSST-1	Toxic shock syndrome toxin 1
UNFPA	United Nations Population Fund
UNHCR	United Nations High Commissioner for Refugees
UNICEF	United Nations Children’s Fund
UTI	Urinary tract infection
